# The Efficiency of Biobased Carbonization Agent and Intumescent Flame Retardant on Flame Retardancy of Biopolymer Composites and Investigation of Their Melt-Spinnability

**DOI:** 10.3390/molecules24081513

**Published:** 2019-04-17

**Authors:** Muhammad Maqsood, Fabian Langensiepen, Gunnar Seide

**Affiliations:** Aachen Maastricht Institute for Biobased Materials, Faculty of Science and Engineering, Maastricht University, Urmonderbaan 22, 6167 RD Geleen, The Netherlands; fabian.langensiepen@maastrichtuniversity.nl (F.L.); gunnar.seide@maastrichtuniversity.nl (G.S.)

**Keywords:** bio-resources, intumescence, melt-spinning, cone calorimetry

## Abstract

The objective of this study is to assess the efficiency of biobased carbonization agent in intumescent formulations (IFRs) to examine the flame retardant properties of polylactic acid (PLA) composites and to investigate their melt-spinnability. We used phosphorous-based halogen free flame retardant (FR) and kraft lignin (KL) as bio-based carbonization agent. After melt compounding and molding into sheets by hot pressing various fire related characteristics of IFR composites were inspected and were characterized by different characterization methods. It was fascinating to discover that the introduction of 5–20 wt% FR increased the limiting oxygen index (LOI) of PLA composites from 20.1% to 23.2–33.5%. The addition of KL with content of 3–5 wt% further increased the LOI up to 36.6–37.8% and also endowed PLA/FR/KL composites with improved anti-dripping properties. Cone calorimetry revealed a 50% reduction in the peak heat release rate of the IFR composites in comparison to 100% PLA and confirmed the development of an intumescent char structure containing residue up to 40%. For comparative study, IFR composites containing pentaerythritol (PER) as a carbonization agent were also prepared and their FR properties were compared. IFR composites were melt spun and mechanical properties of multifilament yarns were tested. The analysis of char residues by energy dispersive X-ray spectrometry (EDS) and SEM images confirmed that PLA/FR/KL composites developed a thicker and more homogeneous char layer with better flame retardant properties confirming that the fire properties of PLA can be enhanced by using KL as a carbonization agent.

## 1. Introduction

Biodegradable polymers from renewable resources have attracted interest due to environmental pollution caused by the disposal of non-degradable polymers derived from finite petroleum reserves [1,2,3,4]. Polylactic acid (PLA) is a biobased thermoplastic polymer obtained from bio-resources and it is progressively replacing oil-based polymers and can be used to develop fire resistant products [5,6]. PLA is less flammable than synthetic thermoplastics such as polyethylene terephthalate (PET), with less visible smoke on burning and a lower peak heat release rate [7,8]. However, PLA is nevertheless combustible which restricts its applications in industry sectors where flame-retardant materials are required [9,10]. 

The fire retardancy of PLA can be enhanced by mixing with inorganic additives containing silicon or phosphorous [11,12,13,14]. Intumescent flame retardant (IFR) systems offer a highly effective strategy to enhance the fire retardancy of PLA because a char structure is developed which acts as a shield between the polymer and heat source hence protecting the polymer material from further burning and dripping [15]. These systems use halogen-free flame retardants (HFFRs) which are not only better for the environment but also more effective [16,17]. IFR systems generally comprise a carbonization agent, an acid source and a blowing agent that produces the char structure. In most of the studies [18,19,20,21] pentaerythritol (PER) a petroleum-based carbonization agent has been used in PLA based IFR systems. Flame retardancy in phosphorous based flame retardants is achieved by the thermal degradation of phosphorous compounds into pyrophosphate and the release of water which eventually dilutes the gas phase, hence the dehydration reaction is catalysed by pyro phosphoric acid [22,23].

Conventional carbonization agents such as PER achieve very low flame retardancy in PLA-based IFR systems [15,19] and different formulations have been therefore developed to overcome this challenge [22,23,24]. For example, PLA-based IFR composites containing PER achieved only a V‑2 value in UL-94 vertical burning tests despite the addition of about 30–40% by weight of the flame-retardant additives, although a combination of PER and APP reduced the dripping behavior of PLA during combustion [25]. To replace PER in PLA-based IFR systems, other researchers have used additives such as starch [19], spirocyclic pentaerythritol bisphosphorate disphosphoryl melamine [22], and graphene [13]. Another potential carbon source is lignin, which is an important component of plant cells and the second most abundant natural material after cellulose [26]. Therefore lignin can be a potential candidate for carbonization agent in IFR systems because it contains phenylpropane repeat units together with aromatic and aliphatic hydroxyl groups [27]. Although the char promoting effect of lignin has been investigated in some studies [26,27,28] and [29,30], not much work has been done related to the melt-spinning of PLA/IFR composites. Cayla et al [31] tried to spin PLA/IFR multifilaments but the mechanical properties of the filaments were too low (5–6 cN·tex^−1^) to be considered for industrial applications. In this study we have tried to optimize the wt% of the additives to improve the FR properties of the composites without compromising the mechanical properties of the multifilaments. 

We therefore investigated the effect of kraft lignin (KL) from wood waste as a carbonization agent in IFR systems as a potential biodegradable substitute for PER together with non-toxic and halogen free flame retardant. The mechanism of intumescence indicating catalytic phosphorylation to produce phosphate esters which eventually dehydrated the lignin and formed char structure containing residue up to 52% has also been discussed in detail. We also tested flammability of different formulations of PLA/APP/KL prepared by melt compounding and then molding into sheets by hot pressing. We characterized the materials by different characterization techniques and measured their fire-retardant properties by conducting UL-94 vertical burning tests, cone calorimetry analysis and by determining the limiting oxygen index. The melt-spinnability of as prepared composites was investigated by producing multifilament yarns on pilot scale melt spinning machine and their mechanical properties were tested.

## 2. Results

### 2.1. Mechanism of Intumescence

**Figure 1:**
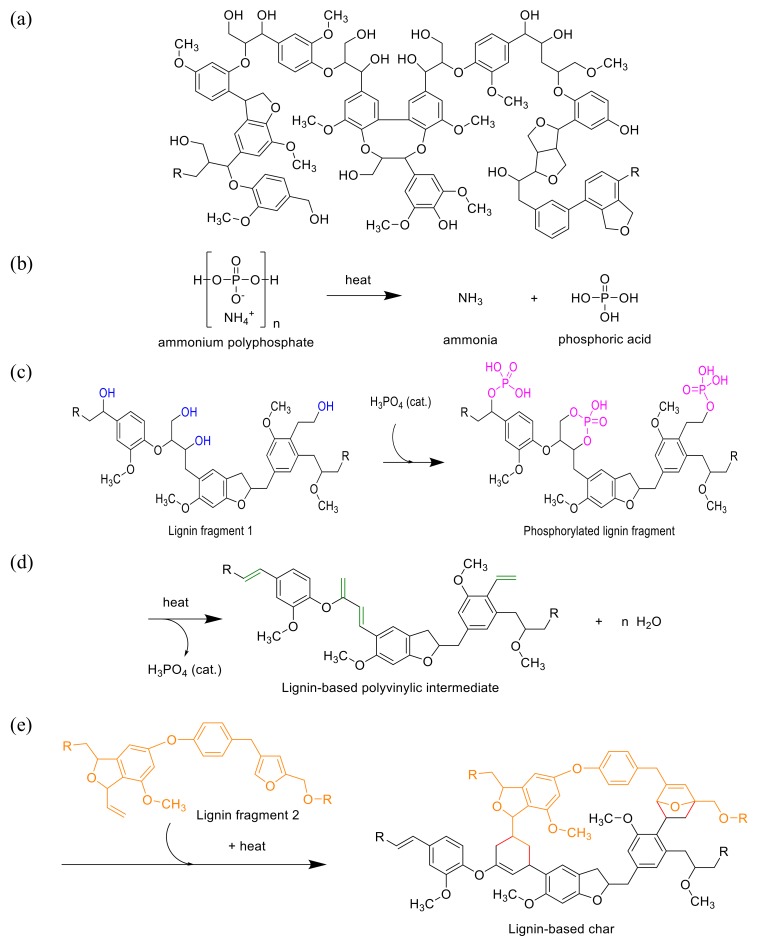
Lignin structure (**a**), Thermal decomposition of ammonium polyphosphate into ammonia and ortho-phosphoric acid (**b**), Catalytic phosphorylation to produce phosphorylated lignin (**c**), Dehydration of lignin and formation of lignin based polyvinylic intermediate (**d**), Diels alder reaction to produce lignin-based char (**e**).

The detailed description of intumescence mechanism was given in our previous article [32] however, in this study chemical reactions showing the mechanism has been discussed. Long chain APP (Form II) was used as flame retardant in PLA polymer. Upon decomposition of APP, phosphoric acid and ammonia was formed. Phosphoric acid acted as acid catalyst in the dehydration process of carbon-based poly-alcohols in lignin. Upon reaction of acid catalyst (phosphoric acid) with alcohol groups in lignin, phosphate esters were formed which were decomposed later to release carbon dioxide and dehydration of lignin was taken place. In the gas phase, the emission of non-flammable carbon dioxide assisted in diluting the oxygen of the air and flammable decomposed products of the material that were burning whereas the resultant char layer in the condensed phase protected the underlying polymeric material from further burning by restricting the free passage of radiant heat and oxygen. This mechanism of intumescence is shown in Figure 1. 

### 2.2. Limiting Oxygen Index and UL-94 Vertical Burning Test

The LOI and UL-94 tests were conducted to assess the flammability of the composites. These flame retardant properties together with the dripping behavior of PLA/APP and PLA/APP/KL IFR composites are mentioned in Table 1. 

100% PLA could not pass UL-94 vertical burning test because it was highly flammable with severe dripping. The LOI of pure PLA was 20.1%. The presence of 5% (*w*/*w*) APP enhanced the LOI of the composite (PLA/APP5) to 23.2% and could only manage to obtain a V-2 rating in UL-94V test. The presence of 10% (*w*/*w*) APP improved the LOI of the composite (PLA/APP10) to 25.7% and managed to obtain a V-1 rating in UL-94V test. When the proportion of APP increased to 15% and 20% (*w*/*w*), the LOI increased to 29.4% (PLA/APP15) and 33.5% (PLA/APP/20) however, both composites managed to achieve only V-1 rating. All of the PLA/APP composites showed evidence of the dripping phenomenon when burning. Although the samples containing PER as carbonization agent improved the LOI to 33.9% and 34.4% by the addition of 3 and 5% (*w*/*w*) of PER but not a significant difference was seen in their UL-94 tests. None of the sample containing PER managed to achieve V-0 rating.

The addition of 3% (*w*/*w*) KL to PLA/APP20 increased the LOI of the new composite (PLA/APP20/KL3) from 33.5% to 36.6% while maintaining the V-0 rating in the UL-94V test. PLA/APP20/KL3 also showed no evidence of dripping when burning. Similar results were observed with higher proportions of KL. The LOI of the composites PLA/APP20/KL5 was 37.8%, and managed to obtain V-0 ratings with no evidence of dripping. These results confirmed that the introduction of KL as a natural carbonization agent increased the LOI of the composites significantly and abolished the melt dripping phenomenon observed in composites lacking KL. All composites containing KL managed to obtain a V-0 rating. 

The UL-94 vertical burning test determines material’s ability to either support or extinguish the flame once it catches fire. The results presented in Table 1 shows no rating of pure PLA to V-1 rating of PLA/APP and V-0 rating of PLA/APP/KL composites. It was observed that the ignition of pure PLA started during the first flame application of 10 s and the burning was continued till the sample was completely burnt up to the sample holding clamp. Although the burning behavior of the sample containing 5% (*w*/*w*) of APP (PLA/APP5) was slightly better to that of pure PLA as the flame extinguished in less than 30 s in each flame application and it achieved V-2 rating but the sample was dripping and ignited the cotton sample placed underneath. Same was the case with 10% (*w*/*w*) of APP (PLA/APP10), although it achieved V-1 rating with slower burning rate compared to PLA/APP5 but it also ignited the cotton sample placed underneath due to dripping. However, with the addition of 15 and 20% (*w*/*w*) of APP (PLA/APP15 and PLA/APP20) the samples achieved better LOI values however, V-1 rating still showed dripping during second application of flame. Furthermore, the flammability of the composites was completely changed by the incorporation of KL in the formulations. The composites containing 3 and 5% (*w*/*w*) of KL were not ignited even after the second application of flame and achieved V-0 rating without dripping in both flame applications. This behavior is attributed to the generation of char layer on specimen’s surface which did not allow the flame to pass through the layer hence the underlying material remained unburnt and restricted the propagation of flame. It can be seen in Table 1 that in comparison to samples containing PER, samples having KL as carbonization agent not only achieved higher LOI% values but also managed to obtain V-0 ratings without dripping phenomenon in UL-94 tests despite of having the same wt% of the additives. 

### 2.3. Cone Calorimetry

The cone calorimeter is equipment used to assess the fire retardancy of a polymeric material and gives useful insight about the flammability of the material. This instrument gives broad information about the combustion behavior of the polymer by measuring parameters such as peak heat release rate (PHRR), time to ignition (TTI), total heat release (THR) and residual mass as a proportion of original mass. Cone calorimetry data for 100% PLA and IFR composites we prepared are summarized in Table 2.

**Figure 2:**
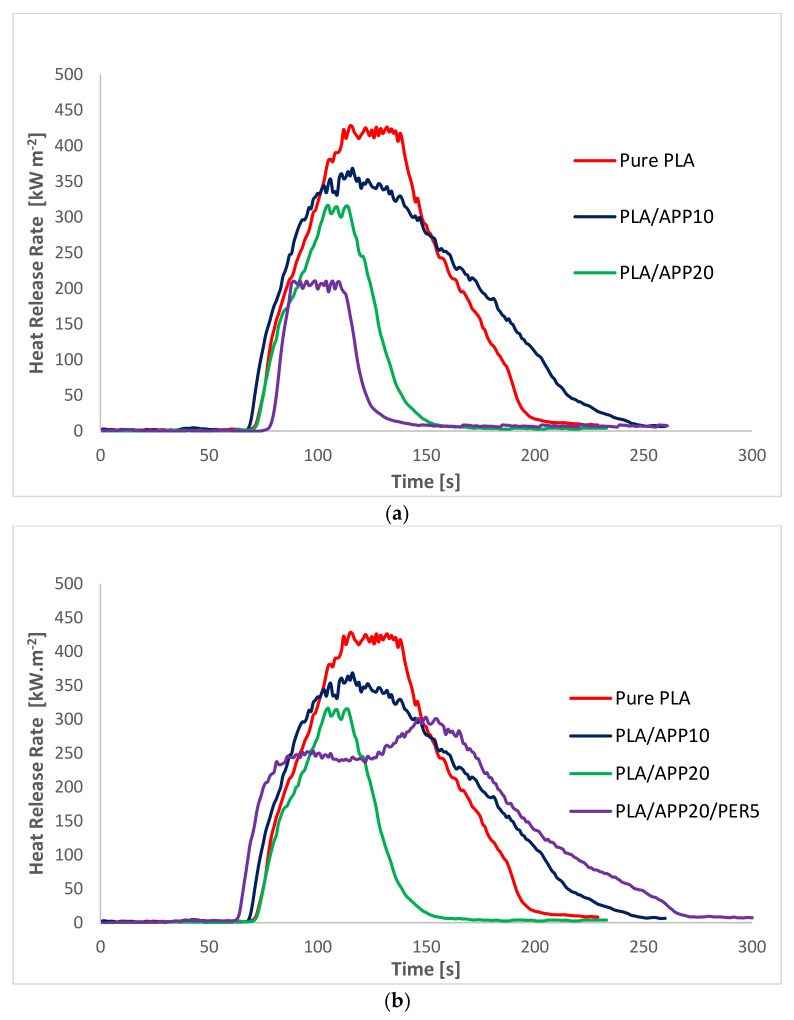
(**a**) Heat release rate curves for pure PLA, PLA/APP10, PLA/APP20 and PLA/APP20/KL5 composites; (**b**) Heat release rate curves for pure PLA, PLA/APP10, PLA/APP20 and PLA/APP20/PER5 composites.

Heat release rate curves for pure PLA, PLA/APP10, PLA/APP20 and PLA/APP20/KL5 are presented in Figure 2a whereas that of PLA/APP20/PER5 is shown in Figure 2b respectively. After ignition pure PLA kept on burning for longer period of time than other samples and therefore, produced a steep curve with a high PHRR (428 kW m^−2^). In contrast, the PHRR of PLA/APP10 and PLA/APP20 were 361 and 316 kW m^−2^ respectively. The addition of PER as carbonization agent in PLA/APP20 composites further reduced the PHRR, as samples containing 5% (*w*/*w*) of PER achieved 300 kW m^−2^. A significant reduction in PHRR was observed when KL was added in PLA/APP20 composites as samples containing 3 and 5% (*w*/*w*) reduced PHRR to 250 and 210 kW m^−2^ respectively. These findings indicated that the combined effect of APP and KL yielded a much thicker char structure after burning, preventing the degradation of the composite by restricting the fire passage to the polymer matrix. It can be seen in Table 2 (and Table S1) and simultaneously in Figure 2a,b that samples containing KL as carbonization agent have much lower PHRR than samples containing PER as carbonization agent despite of having same wt% in PLA/APP20 composites.

**Figure 3:**
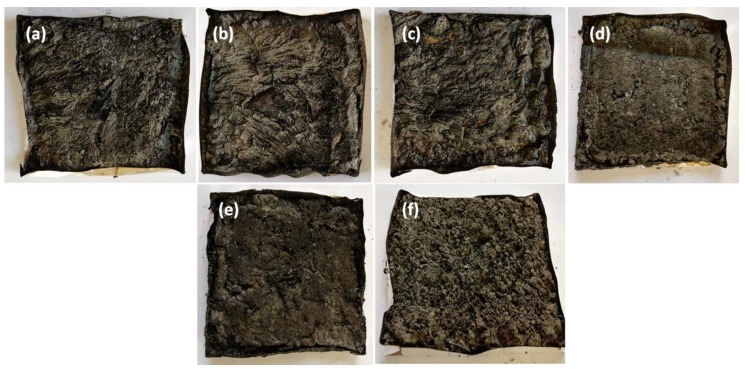
Char residues after cone calorimetry: PLA/APP5 (**a**), PLA/APP10 (**b**), PLA/APP15 (**c**), PLA/APP20 (**d**), PLA/APP20/KL3 (**e**), and PLA/APP20/KL5 (**g**).

**Figure 4:**
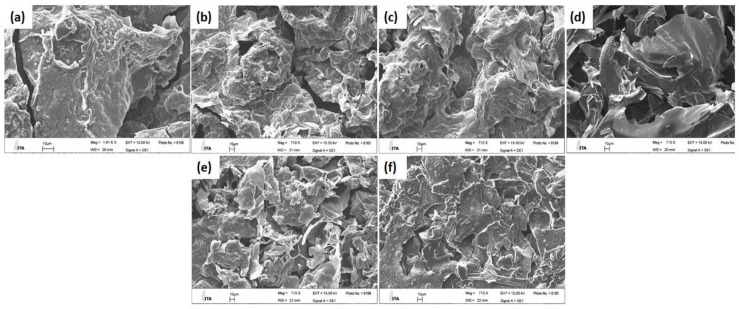
SEM images of the char residues of PLA/APP5 (**a**), PLA/APP10 (**b**), PLA/APP15 (**c**), PLA/APP20 (**d**), PLA/APP20/KL3 (**e**), and PLA/APP20/KL5 (**f**) after cone calorimetry test Scale bar in all panels = 10 μm, Magnification = 710×.

Figure 2a demonstrates that the heat release rate of the composites containing APP alone (PLA/APP10, PLA/APP20) and together with KL (PLA/APP20/KL5) changed quite dramatically in comparison to pure PLA (428 kW m^−2^). The production of intumescent char in case of samples containing APP alone was delayed due to the emission of volatile compounds and the degree of swelling in the char was also reduced, hence a porous char structure was formed as shown in Figure 3 and Figure 4. 

Figure 3 demonstrates images of the residual samples after conducting cone calorimetry test. The char residues of PLA/APP5, PLA/APP10, PLA/APP15 and PLA/APP20 were loosely bound, and the structure in each case was porous and discontinuous due to insufficient char formation as shown by the SEM images of char residues in Figure 4. Heat and mass transfer therefore could not be inhibited effectively in these composites. In contrast, the samples containing KL (particularly PLA/APP20/KL5) produced a more compact char with a dense and uniform structure, reducing fuel and heat transfer to inhibit combustion and prevent further burning of the underlying polymeric substrate. 

The barrier effectiveness and shielding efficiency of a residue is identified by the compactness of a char structure. The char structures of the residues improved with increasing APP content however the char formed was loosely bound and porous due to non-cohesion of the agglomerates. Furthermore, the char structures of residues of the composites containing APP and KL were stable, more uniform and compact due to cohesion of the agglomerates. KL particles were supposed to fill the empty spaces between the APP particles which densified the agglomerates of the residues hence more stable, uniform and compact char structures were produced for the samples containing KL. The thickness of the samples containing lignin also increased dramatically due to char formation after burning, from an initial thickness of 3 mm to approximately 1–1.5 cm.

Table 2 shows that the TTI of 100% PLA was 63 s, but when mixed with 20% (*w*/*w*) APP in the composite PLA/APP20, the TTI increased to 76 s. The samples containing PER as carbonization agent also had similar TTI values to what we get with samples containing KL as carbonization agent. The ignition of a material is typically dependent on the concentration of pyrolysis gases, which are released when a material is degraded until the concentration reaches a value that ignition is supported. A long TTI therefore reflects slower decomposition mainly due to the presence of APP and KL. 

**Figure 5:**
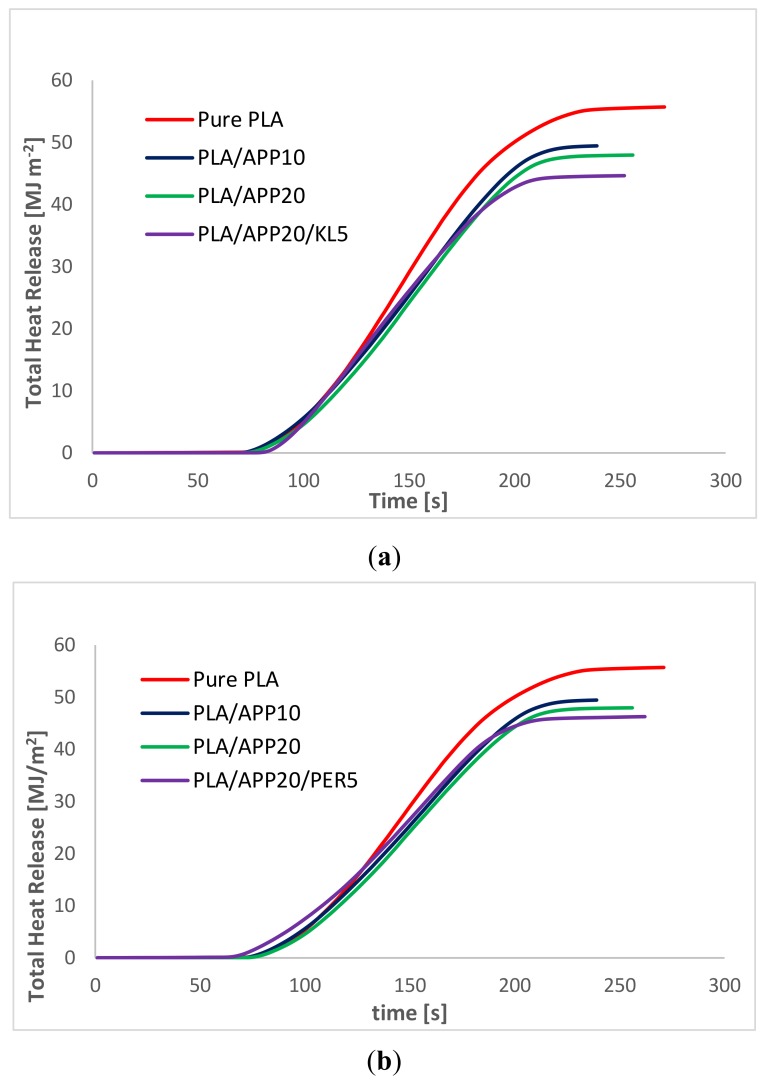
(**a**) Total heat release curves of 100% PLA, PLA/APP and PLA/APP/KL composites; (**b**) Total heat release curves of 100% PLA, PLA/APP and PLA/APP/PER composites.

Figure 5a demonstrates the THR curves of 100% PLA and the PLA/APP and PLA/APP/KL composites. The THR of 100% PLA was 55.7 MJ m^−2^ whereas the values for PLA/APP20 and PLA/APP20/KL5 were 47.9 and 44.6 MJ m^−2^, respectively. This indicates that both PLA/APP20 and PLA/APP20/KL5 reduced the total quantity of fuel accessible for burning, which confirms the superior fire retardant performance of these composites. Figure 5b shows the THR curves of PLA/APP20/PER composites as a comparison to THR curves of PLA/APP20/KL composites in Figure 5a. THR value for PLA/APP20/PER5 composite was 46.4 MJ m^−2^ in comparison to 44.6 MJ m^−2^ for PLA/APP20/KL5 composite. The combination of KL and APP makes the composites more flame resistant, and the production of intumescent char on the matrix surface introduced a layer of thermal insulation between the flame and the surface of the material, which extinguished the flame by preventing contact with combustible gases as well as oxygen. The high concentrations of APP and KL diluted the polymer matrix very strongly and there was not much material available to continue the burning process. The thermal decomposition process led to the dehydration of APP and water vapors released in due course cooled down the gas phase and accessible fuel for combustion was diluted therefore, the total heat release (THR) was decreased with increasing APP content. Due to the endothermic decomposition reaction of APP the heating of the condensed phase was also reduced. THR was further reduced by the addition of KL as the emission of pyrolysis gases were decelerated by the formation of char layer which not only provided the physical barrier to the emission of pyrolysis gases but also enhanced heat shielding effect. The hypothesis of this study is also in good relation with the THR as the previous studies [21,33,34] done with PLA in relation with other carbonization agents had much higher THR than what we achieved in this study.

**Figure 6:**
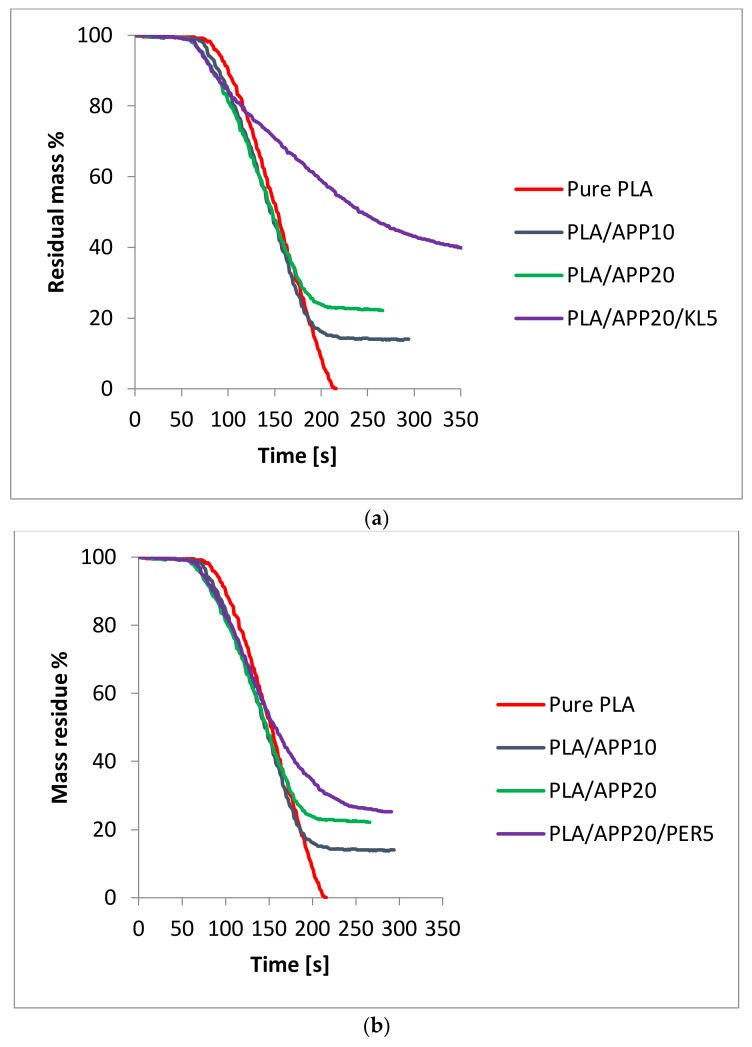
(**a**) Residual mass (percentage of original mass) of 100% PLA, PLA/APP and PLA/APP/KL composites; (**b**) Residual mass (percentage of original mass) of 100% PLA, PLA/APP and PLA/APP/PER composites.

Figure 6a shows the residual mass curves for 100% PLA and the PLA/APP and PLA/APP/KL composites as a percentage of the original mass. The residual mass of PLA/APP20/KL5 was 40%, much higher than that of PLA/APP20 (22%), and the residual mass of pure PLA was close to 0%. Figure 6b shows the residual mass curves of PLA/APP20/PER composites as a comparison to residual mass curves of PLA/APP20/KL composites in Figure 6a. The residual mass% for PLA/APP20/PER5 was 25% in comparison to 40% for PLA/APP20/KL5 composites (Table S3). The higher residual mass correlated with the production of more char, which in turn reflects the lower THR values. The greater residual mass also reflects in greater char formation due to the combined effect of acid and carbonization agent. The residual mass% achieved in this study is also higher than the residual mass% of other studies done with PLA in relation to other carbonization agents [35,36].

### 2.4. Energy Dispersive X-ray Spectrometry

EDS spectrometry was used to inspect the composition of elements present in the char residues. The wt% of the elements present in char residues are compared in Table 3. 

It can be seen in Table 3 that PLA/APP20 contained highest wt% of Oxygen (40.9%) and least wt% of Carbon (21.2%) in char residues. KL favored charring as proved by the increase of C content and the decrease of O content. It is likely that the main part of P remains in the residue (high value of P content). Since the char content increases with increasing KL content, the P content in the char residue necessarily decreases. However, P content is over estimated and quantitative EDS must be performed on flat sample. Moreover, the phosphate compounds developed by the reaction of APP-KL enhanced the char production rate because they stayed in the condensed phase. The increment in the Carbon content (wt%) of PLA/APP/KL residues is due to an increased char formation by the addition of KL.

### 2.5. Scanning Electron Microscopy

**Figure 7:**
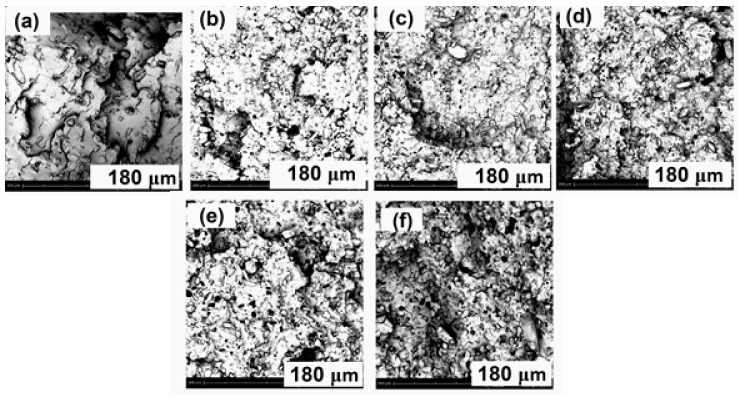
SEM images of composites PLA/APP5 (**a**), PLA/APP10 (**b**), PLA/APP15 (**c**), PLA/APP20 (**d**), PLA/APP20/KL3 (**e**), and PLA/APP20/KL5 (**f**). Scale bar in all panels = 180 μm, Magnification = 710×.

The dispersion of different proportions of APP and KL in the PLA matrix was investigated by scanning electron microscopy to characterize the distribution of the additives, given that a uniform distribution achieves better fire retardant properties. Figure 7 shows images of the composites PLA/APP5, PLA/APP10, PLA/APP15, PLA/APP20, PLA/APP20/KL3 and PLA/APP20/KL5. APP and KL particles of different sizes and shapes, and showing different levels of interfacial adhesion with the PLA matrix, were incorporated successfully. In the PLA/APP formulations lacking KL, the additive was dispersed uniformly regardless of its proportion in the mixture (5–20% *w*/*w*), indicating the uniform mixing of APP with the substrate during sample preparation. However, weak interfacial bonding between PLA and APP was apparent based on the appearance of small holes during fracturing. Nevertheless, all images confirmed that APP and KL has been incorporated successfully into the PLA matrix.

### 2.6. Thermogravimetric Analysis

**Figure 8:**
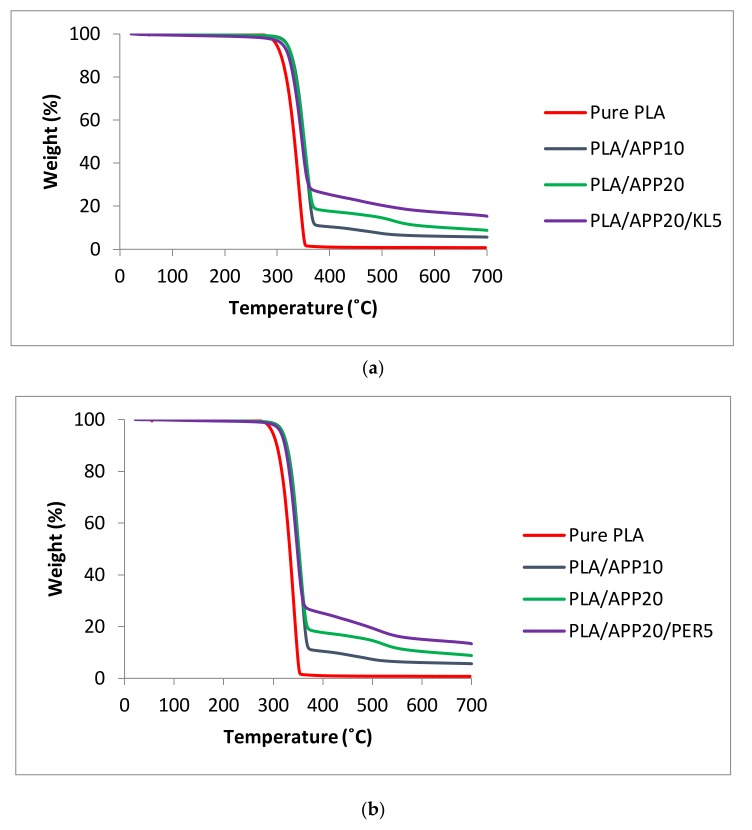
(**a**) Thermogravimetric curves of 100% PLA, PLA/APP and PLA/APP/KL composites; (**b**) Thermogravimetric curves of 100% PLA, PLA/APP and PLA/APP/KL composites.

The thermal decomposition and thermal stability of the polymers was assessed by thermogravimetric analysis and the residual mass of the samples was determined at 700 °C. The thermogravimetric behavior of pure PLA and of the IFR composites was expressed as the temperature corresponding to 5% and 50% weight loss in a nitrogen atmosphere (T5 and T50, respectively) as shown in Table 4 (and Table S2), and the corresponding thermogravimetric curves (Figure 8a,b). The decomposition of PLA/APP5 started at 310 °C and 50% loss was recorded at 343 °C. The residue at 700 °C represented 3.54% of the weight of the original sample. PLA/APP10 showed a similar performance but with slightly higher T5 and T50 values and a residual weight of 5.63%. The trend continued for PLA/APP15 and PLA/APP20, with marginal further increases in the T5 and T50 values and residual weights of 8.16% and 8.78%, respectively. The introduction of KL enhanced the thermal stability of the composites even further. The T5 and T50 values of PLA/APP20/KL3 were higher than those of any composite without KL, and the residual weight increased to 13.34%. As more KL was incorporated, the T5 and T50 values increased further and the residual weight was 15.32% for PLA/APP20/KL5. 

Thermogravimetric curves of 100% PLA, PLA/APP10, PLA/APP20 and PLA/APP20/KL5 show the residual weight as a function of temperature, up to 700 °C (Figure 8a). Figure 8b shows the residual weight% for TG curves of PLA/APP20/PER composites as a comparison to residual weight% for TG curves of PLA/APP20/KL composites in Figure 8a. The residual weight% for PLA/APP20/PER5 composite was 13.20 in comparison to 15.32 for PLA/APP20/KL5. The curves indicate that most of the thermal decomposition occurs between 300 °C and 400 °C and that pure PLA decomposes at a lower temperature than all the composites. Whereas the composites all degrade within a narrow temperature window, increasing the concentration of APP causes more residual weight to remain at temperatures between 375 °C and 700 °C, and adding KL at increasing concentrations has a further, additive effect. The thermal stabilities of the composites containing KL are therefore better than those of composites containing APP alone or composites containing PER. 

## 3. Discussion

The addition of APP in the concentration range of 5% (*w*/*w*) to 20% (*w*/*w*) enhanced the LOI from 20% (pure PLA) to 33.5% (PLA/APP20) (Table 1). With an increasing amount of APP, a higher percentage of oxygen is required in order to sustain ignition of the sample. This is due to the fuel dilution in the gas phase by the discharge of water vapor and NH_3_ as a result of dehydration of APP. The addition of KL in the formulations (3%, and 5%) not only increased the LOI% of the samples but also increased the mass residue. Moreover, the increased amount of residue establishment is suggested to enhance the shielding of the samples against heat and to slow down the pyrolysis process by acting as a barrier against the emission of pyrolysis gases. Therefore, the emission of fuel in the gas phase is minimized by the addition of APP. 

Hence these results are in good relation with the main hypothesis of this study since in previous studies [25,30] the use of PER as carbonization agent could only achieve V-2 rating despite the addition of 30 to 40% (*w*/*w*) in the polymer matrix, whereas in this study V-0 rating was achieved at much lower loading% (*w*/*w*) due to the combined effect of APP and KL. 

In intumescent system flame retardancy is achieved by the swelling of the substrate in the condensed phase (as explained in mechanism of intumescence) which generates a sponge like multicellular structure called char which shields the fundamental material from heat transfer to the sample. Moreover, the char structure also provides shielding against fuel and heat transfer from the condensed phase to the sight of burning. 

The weaker intumescence or a porous char structure in the samples containing APP alone is caused by decreased viscosity in the condensed phase. The production of a uniform and compact char structure is mainly dependent on the viscosity of the sample in condensed phase. The lower viscosity in the condensed phase released the water vapors via bubbling which were produced by the dehydration of APP and hence no longer available for the swelling of the char structure. If the viscosity in the condensed phase is too low then APP alone cannot generate enough pressure for the swelling of the substrate. Due to this porous char structure both fuel gases (volatile compounds) and water vapors could easily pass through the unclosed cells, therefore the HRR of the samples containing APP alone is higher in comparison to HRR of the samples containing APP and KL. The reason is that the combined effect of acid (APP) and carbon source (KL) produced more compact char structure which hindered the discharge of fuel gases and water vapors which ultimately increased the viscosity of the condensed phase and a result more swelling of the char was observed. Therefore, the combined effect of APP and KL further decreased the PHRR down to 210 kW m^−2^ which is 50% less than pure PLA. The hypothesis of this study also had a positive influence on the heat emitted during combustion process as HRR in this study is much lower to that we witnessed in other studies done with PLA using other carbonization agents [9,11,12,13,14].

The higher TTI of the samples is predominantly attributed to the lower emission of pyrolysis gases which serve as a fuel material for the flame. Therefore, a uniform and compact char structure will be required which can hinder the diffusion of pyrolysis gases from the melt substrate to the sight of burning. But the lower TTI of the samples containing APP alone is mainly due to higher emission of pyrolysis gases which are caused by weaker swelling of the substrate which generates porous char structure with unclosed cells provides empty spaces for the pyrolysis gases to escape and serve as a fuel source to the flame. However, with the combined effect of APP and KL the melt viscosity of the substrate increased which improved the swelling of the substrate which generates more compact char structure thereby hindering the free escape of pyrolysis gases hence the ignition time is prolonged.

The lower mechanical properties of multifilament yarns containing KL and APP are mainly due to lower interfacial adhesion between the components and large parts of additives with relative low aspect ratio. It is more likely, that the large particles acted as starting point of failure during loading. Another reason could be as the lignin content was increased, hydroxyl groups present in lignin formed hydrogen bonds with PLA substrate, as a result molecular chain gets more entangled which created free void spaces in the multifilament yarns by restricting molecular chain mobility, hence their elongation at break reduced and they broke at much lower force compared to pure PLA as explained by Cayla et al. [31]. 

## 4. Materials and Methods

### 4.1. Materials

Granular PLA resin (Luminy L130) was attained from Total-Corbion (Gorinchem, Netherlands). Non-halogenated flame retardant Exolit APP 422, a fine-particle APP containing 14% *w*/*w* nitrogen and 31% *w*/*w* phosphorous (decomposition temperature > 275 °C), was obtained from Clariant Plastics & Coatings SA (Louvain-la-Neuve, Belgium). Exolit APP 422 is insoluble in water and thermally stable at higher temperatures due to long chains (*n* > 1000) and has been used as acid donor in this study. The kraft lignin powder “UPM BioPiva 100” was purchased from UPM Biochemicals (Helsinki, Finland). Pentaerythritol (PER) was obtained from Acros (Molinons, France). PLA, APP, PER and KL were vacuum dried at 100 °C for 6 h before compounding. 

### 4.2. Preparation of PLA/IFR Composites

Coperion twin screw compounder was used to prepare PLA/APP, PLA/APP/PER and PLA/APP/KL composites at 190 °C. In the first phase, PLA/APP composites with an APP content of 5%, 10%, 15% and 20% (*w*/*w*) were compounded at screw rotation speed of 150 rpm and were entitled PLA/APP5, PLA/APP10, PLA/APP15 and PLA/APP20, respectively. The temperatures of the three heating zones were kept at 175 °C, 180 °C and 185 °C, respectively. The extrudate was cut into pellets. In the second phase, PLA/APP20 pellets (APP content = 20% *w*/*w*) with a KL content of 3%, and 5% (*w*/*w*) were compounded at screw rotation speed of 200 rpm and were named PLA/APP20/KL3 and PLA/APP20/KL5, respectively. PLA/APP20 pellets were dosed in the first feeding zone whereas KL was fed in the second feeding zone to ensure proper mixing. The same procedure was adopted for PLA/APP20/PER3 and PLA/APP20/PER5 composites. Sheets of the as prepared composites were produced by compression molding at 190 °C for the subsequent testing along with sheets of pure PLA for comparison. 

### 4.3. Thermogravimetric Analysis

Thermogravimetric behavior of the composites as well as of 100% PLA was assessed using a TGA Q5000 from TA Instruments. The specimens (10–15 mg) were heated at a constant rate of 10 °C min^−1^ up to 700 °C under nitrogen at a flow rate of 50 mL min^−1^. The thermal decomposition temperature and the temperature at which maximum degradation took place were calculated along with the residual percentage of the sample compared to the initial mass. The thermogravimetric curves of specimens were plotted after analysis.

### 4.4. Scanning Electron Microscopy

The surface morphology of IFR composites, inspection of additives dispersion in the PLA matrix and energy dispersive X-ray spectrometry (EDS) was done by scanning electron microscopy using a Hitachi TM-1000 device (Chiyoda, Tokyo, Japan). Strands of IFR composites were fractured after dipping in liquid nitrogen and later on gold sputtering was done to produce a conductive surface prior to analysis.

### 4.5. Limiting Oxygen Index and UL-94 Vertical Burning Tests

The limiting oxygen index (LOI) is known as the fraction of oxygen necessary to facilitate the burning of a test sample, so high LOI values indicate low flammability. The LOI test was conducted using a Stanton Redcroft instrument (Thermal Sciences, Mansfield, MA, USA) by placing samples (100 mm × 10 mm × 3 mm) vertically in a glass column and supplying a combination of oxygen and nitrogen gas. The specimens were burned with the flame pointing downwards to the non-burnt part according to standard test method ISO 4589. 

The UL-94 test classifies materials according to their ability to promote or inhibit the spread of fire. UL-94 vertical burning tests were conducted by suspending specimens (100 mm × 10 mm × 3 mm) vertically and applying a flame to the lower surface according to ISO 9773. Samples that demonstrate self-extinguishing behavior and that do not drip after burning are ranked highest in the classification (V-0).

### 4.6. Cone Calorimetry Test

Cone calorimetry tests were conducted by placing samples (100 mm × 100 mm × 3 mm) in a Stanton Redcroft instrument (Thermal Sciences) and exposing them to a heat flux of 35 kW m^−2^ according to a standard test method ISO 5660. We recorded important flammability parameters and mass residue as a proportion of initial sample weight.

### 4.7. Mechanical Testing of Multifilament Yarns

The tenacity and elongation at break of multifilaments were tested on Zwick Roell testing machine by using EN ISO 5079 standard method. The specimen lengths (50 mm) and rate of deformation (50 mm min^−1^) were kept constant for all samples. Ten specimens were prepared from each sample and their average results with standard deviations were recorded.

### 4.8. Melt Spinning of IFR Composites

**Figure 9:**
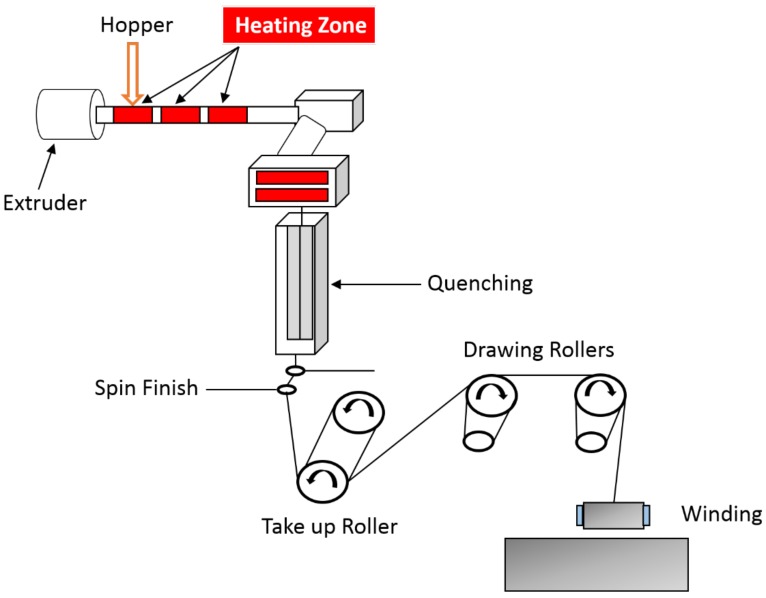
Schematic diagram of pilot scale melt spinning machine.

IFR composites were melt spun using Fourne Maschinenbau GmbH (Impekoven, Germany) pilot scale melt spinning machine. Pellets were first fed into a hopper and then transported to a single screw extruder where they were melted at a temperature range of 195 °C to 220 °C. The melted material was then injected in a spinneret die of 1.2 mm diameter each, with the help of spinning pump rotating at constant revolutions per minute ensuring a homogeneous flow of the material. These single filaments coming out of the spinneret were then cooled at 18 °C by maintaining the cool air velocity of 0.5 m·s^−1^ and then combined together to multifilaments by applying a spin finish. The multifilaments were collected on a take up roller rotating at 450 m/min and then hot drawn between two set of rollers rotating at varying speeds. The speeds of the first and second set of heated rollers were maintained at 550 m/min and 650 m/min respectively ensuring a draw ratio of 1.4 (maximum possible draw ratio). The multi-filaments were then winded on the winder at 650 m/min. A schematic diagram of pilot scale melt spinning machine is shown in Figure 9. 

Composites were melt spun and it was observed that as the loading content of APP and KL was increased, the multifilament yarns were not able to withstand the same draw ratio which was applied for pure PLA. For other compositions of composites, draw ratio was reduced gradually from 2 to 1.4 in order to spin the composites without breakage. This reduction in draw ratio resulted in lower mechanical properties of multifilament yarns produced from these composites predominantly due to amorphous nature with little or no crystallinity induced in the filament structure.

The tenacity and elongation at break of multifilament yarns are mainly influenced by the wt% (*w*/*w*) of the additives incorporated in the PLA matrix therefore the mechanical properties of the multifilament yarns containing higher amount of APP, PER and KL were on the lower side (Table 5) than that of multifilament yarns produced from pure PLA. 

## 5. Conclusions

In this study, the efficiency of KL as a biobased carbonization agent in intumescent formulations was assessed and compared with conventional carbonization agent (PER) to examine the flame retardant properties of PLA/APP/KL composites. The mechanism of intumescence and melt-spinnability of IFR composites was also investigated. IFR composites comprising different formulations were produced with and without carbonization agents (KL and PER) by melt extrusion and their flammability was assessed by UL-94, LOI and cone calorimetry tests. The introduction of 5–20% (*w*/*w*) APP improved the LOI from 20.1 (pure PLA) to 23.2–33.5% for the composites, but the further addition of 5% (*w*/*w*) KL (PLA/APP20/KL5) increased the LOI to 37.8% and the composite achieved a V-0 value in UL-94 test with no dripping. In comparison, sample containing 5wt% of PER achieved LOI only up to 34.4% with V-1 rating moreover dripping phenomenon was observed in second flame application. The PHRR and THR of the composites containing KL were significantly lower than the equivalent values for composites containing PER. A remarkably low PHRR was observed for PLA/APP20/KL5 (210 kW m^−2^) which is 50% less than the PHRR of 100% PLA. The occurrence of 20% APP increased the TTI to 76 s and the further addition of 5% KL increased the TTI to 81 s however, the TTI for PER composites were almost in the same range to that of KL composites. The presence of APP and KL also made IFR composites more thermally stable. Thermogravimetric curves showed that whereas PLA left almost no residual weight after heating to 700 °C, the PLA/APP20 composite produced 8.78% residual mass and this increased to 15.32% for composite PLA/APP20/KL5. In comparison a composite containing 5wt% of PER (PLA/APP20/PER5) could only achieve a residual mass up to 13.20%, confirming that PLA/APP/KL composites are more thermally stable. The analysis of char residues by energy dispersive X-ray spectrometry (EDS) and SEM images confirmed that PLA/FR/KL composites developed a thicker and more homogeneous char layer with better flame retardant properties confirming that the fire properties of PLA can be enhanced by using KL as a carbonization agent. 

## Figures and Tables

**Table 1:** Fire retardant properties of 100% PLA, the PLA/APP and PLA/APP/KL IFR composites.

No	Formulations	PLA % (*w*/*w*)	APP % (*w*/*w*)	KL % (*w*/*w*)	LOI%	UL-94	Dripping
1	PLA	100	0	0	20.1	Failed	Y/Y
2	PLA/APP5	95	5	0	23.2	V-2	Y/Y
3	PLA/APP10	90	10	0	25.7	V-1	Y/Y
4	PLA/APP15	85	15	0	29.4	V-1	N/Y
5	PLA/APP20	80	20	0	33.5	V-1	N/Y
6	PLA/APP20/PER3	77	20	3	33.9	V-1	N/Y
7	PLA/APP20/PER5	75	20	5	34.4	V-1	N/Y
8	PLA/APP20/KL3	77	20	3	36.6	V-0	N/N
9	PLA/APP20/KL5	75	20	5	37.8	V-0	N/N

PLA = Polylactic acid, APP = Ammonium polyphosphate, KL = Kraft lignin, LOI = Limiting Oxygen Index, N/Y corresponds to NO/YES for dripping in first/second flame application.

**Table 2:** Cone calorimetry data for 100% PLA, and PLA/APP and PLA/APP/KL IFR composites.

Formulation	TTI (s)	PHRR (kW m^−2^)	THR (MJ·m^−2^)	Residual Mass (%)	TSP (m^2^·m^−2^)	EHC (kJ·g^−1^)
PLA	63 ± 1.1	428 ± 7	55.7 ± 0.27	0 ± 0.00	43 ± 2	17.33 ± 1.60
PLA/APP5	66 ± 1.4	382 ± 6	52.1 ± 0.16	9 ± 0.03	274 ± 13	16.10 ± 0.90
PLA/APP10	69 ± 2.3	361 ± 3	49.7 ± 0.22	14 ± 0.01	230 ± 17	16.05 ± 1.22
PLA/APP15	72 ± 1.8	336 ± 2	49.1 ± 0.17	17 ± 0.09	190 ± 19	15.93 ± 1.13
PLA/APP20	76 ± 2.8	316 ± 8	47.9 ± 0.31	22 ± 0.04	164 ± 9	15.46 ± 0.86
PLA/APP20/PER3	78 ± 3.4	310 ± 9	47.5 ± 0.41	23 ± 0.05	221 ± 23	15.13 ± 1.56
PLA/APP20/PER5	80 ± 2.9	300 ± 7	46.4 ± 0.29	25 ± 0.07	209 ± 17	14.62 ± 1.34
PLA/APP20/KL3	79 ± 1.1	250 ± 4	45.0 ± 0.29	25 ± 0.05	155 ± 21	12.68 ± 1.43
PLA/APP20/KL5	81 ± 1.4	210 ± 6	44.6 ± 0.12	40 ± 0.03	103 ± 14	11.72 ± 1.18

TTI = time to ignition; PHRR = peak heat release rate; THR = total heat release; TSP = Total smoke production; EHC = Effective heat of combustion.

**Table 3:** Elemental analysis of char residues.

No.	Samples	C (wt %)	O (wt %)	P (wt %)	Al (wt %)
1	PLA/APP20	21.2	40.9	34.6	3.3
2	PLA/APP20/KL3	34.9	38.0	26.6	0.5
3	PLA/APP20/KL5	37.7	34.2	26.4	1.7

**Table 4:** Thermogravimetric analysis of pure PLA and the PLA/APP and PLA/APP/KL composites.

No	Formulations	T_5_ (°C)	T_50_ (°C)	Residue at 700 °C (%, *w*/*w*)
1	PLA	298	333	0
2	PLA/APP5	310	343	3.54
3	PLA/APP10	318	351	5.63
4	PLA/APP15	320	355	8.16
5	PLA/APP20	328	358	8.78
6	PLA/APP20/PER3	329	360	10.63
7	PLA/APP20/PER5	328	365	13.20
8	PLA/APP20/KL3	330	362	13.34
9	PLA/APP20/KL5	330	365	15.32

T_5_ = 5% weight loss, T_50_ = 50% weight loss

**Table 5:** Mechanical properties of pure PLA, PLA/APP and PLA/APP/KL multifilament yarns.

Formulations	Tenacity ± (cN/tex)	Elongation at Break ± (%)
PLA	17.88 ± 6	123.11 ± 23
PLA/APP5	14.44 ± 4	94.73 ± 21
PLA/APP10	13.19 ± 7	80.66 ± 14
PLA/APP15	10.37 ± 8	64.97 ± 13
PLA/APP20	9.81 ± 6	58.16 ± 24
PLA/APP20/PER3	9.33 ± 7	55.43 ± 12
PLA/APP20/PER5	8.10 ± 5	49.56 ± 17
PLA/APP20/KL3	8.76 ± 3	50.12 ± 11
PLA/APP20/KL5	7.43 ± 4	45.25 ± 19

## Supplementary Materials

The following are available online, Table S1: supplementary data after cone calorimetry test, Table S2: supplementary data for TGA curves, Table S3: supplementary data for mass residue%.

## References

[1] Al-itry R., Lamnawar K., Maazouz A. (2012). Improvement of thermal stability, rheological and mechanical properties of PLA, PBAT and their blends by reactive extrusion with functionalized epoxy. Polym. Degrad. Stab..

[2] Hussain T., Tausif M., Ashraf M. (2015). A review of progress in the dyeing of eco-friendly aliphatic polyester- based polylactic acid fabrics. J. Clean. Prod..

[3] Armentano I., Bitinis N., Fortunati E., Mattioli S., Rescignano N., Verdejo R., Lopez-manchado M.A., Kenny J.M. (2013). Multifunctional nanostructured PLA materials for packaging and tissue engineering. Prog. Polym. Sci..

[4] Martin J.A. (2014). An effect of lactic acid oligomers on the barrier properties of polylactide. J. Mater. Sci..

[5] Cheng K.C. (2015). Flammability and tensile properties of polylactide nanocomposites with short carbon fibers. J. Mater. Sci..

[6] Nofar M., Park C.B. (2014). Poly (lactic acid) foaming. Prog. Polym. Sci..

[7] Cheng X., Guan J., Tang R., Liu K. (2015). Improvement of flame retardancy of poly (lactic acid) nonwoven fabric with a phosphorus- containing flame retardant. J. Ind. Text..

[8] Rhim J., Park H., Ha C. (2013). Bio-nanocomposites for food packaging applications. Prog. Polym. Sci..

[9] Fukushima K., Murariu M., Camino G., Dubois P. (2010). Effect of expanded graphite/layered-silicate clay on thermal, mechanical and fire retardant properties of poly(lactic acid). Polym. Degrad. Stab..

[10] Uddin F. (2016). Flame-retardant fibrous materials in an aircraft. J. Ind. Text..

[11] Lin H.J., Liu S.R., Han L.J., Wang X.M., Bian Y.J., Dong L.S. (2013). Effect of a phosphorus-containing oligomer on flame-retardant, rheological and mechanical properties of poly (lactic acid). Polym. Degrad. Stab..

[12] Lin H., Han L., Dong L. (2014). Thermal degradation behavior and gas phase flame-retardant mechanism of polylactide/PCPP blends. J. Appl. Polym. Sci..

[13] Mngomezulu M.E., Luyt A.S., John M.J. (2017). Morphology, thermal and dynamic mechanical properties of poly(lactic acid)/expandable graphite (PLA/EG) flame retardant composites. J. Thermoplast. Compos. Mater..

[14] Murariu M., Bonnaud L., Yoann P., Fontaine G., Bourbigot S., Dubois P. (2010). New trends in polylactide (PLA)-based materials: “Green” PLA-Calcium sulfate (nano)composites tailored with flame retardant properties. Polym. Degrad. Stab..

[15] Atabek Savas L., Mutlu A., Dike A.S., Tayfun U., Dogan M. (2017). Effect of carbon fiber amount and length on flame retardant and mechanical properties of intumescent polypropylene composites. J. Compos. Mater..

[16] Depeng L., Chixiang L., Xiulei J., Tao L., Ling Z. (2017). Synergistic effects of intumescent flame retardant and nano-CaCO 3 on foamability and flame-retardant property of polypropylene composites foams. J. Cell. Plast..

[17] Duquesne S., Samyn F., Ouagne P., Bourbigot S. (2015). Flame retardancy and mechanical properties of flax reinforced woven for composite applications. J. Ind. Text..

[18] Wang D.Y., Leuteritz A., Wang Y.Z., Wagenknecht U., Heinrich G. (2010). Preparation and burning behaviors of flame retarding biodegradable poly(lactic acid) nanocomposite based on zinc aluminum layered double hydroxide. Polym. Degrad. Stab..

[19] Wang J., Ren Q., Zheng W., Zhai W. (2014). Improved flame-retardant properties of poly(lactic acid) foams using starch as a natural charring agent. Ind. Eng. Chem. Res..

[20] Wang K., Wang J., Zhao D., Zhai W. (2017). Preparation of microcellular poly(lactic acid) composites foams with improved flame retardancy. J. Cell. Plast..

[21] Qian Y., Wei P., Jiang P., Li Z., Yan Y., Ji K. (2013). Aluminated mesoporous silica as novel high-effective flame retardant in polylactide. Compos. Sci. Technol..

[22] Zhan J., Song L., Nie S., Hu Y. (2009). Combustion properties and thermal degradation behavior of polylactide with an effective intumescent flame retardant. Polym. Degrad. Stab..

[23] Wang D.Y., Song Y.P., Lin L., Wang X.L., Wang Y.Z. (2011). A novel phosphorus-containing poly(lactic acid) toward its flame retardation. Polymer.

[24] Zhang R., Xiao X., Tai Q., Huang H., Yang J., Hu Y. (2012). Preparation of lignin–silica hybrids and its application in intumescent flame-retardant poly(lactic acid) system. High Perform. Polym..

[25] Bourbigot S., Duquesne S., Fontaine G., Bellayer S., Turf T., Samyn F. (2008). Characterization and Reaction to Fire of Polymer Nanocomposites with and without Conventional Flame Retardants. Mol. Cryst. Liq. Cryst..

[26] Reti C., Casetta M., Duquesne S., Bourbigot S., Delobel R. (2006). Flammability properties of intumescent PLA including starch and lignin. Polym. Adv. Technol..

[27] Zhang R., Xiao X., Tai Q., Huang H., Hu Y. (2012). Modification of lignin and its applications as a char agent in intumescent flame retardant polylactic acid. Polym. Eng. Sci..

[28] Costes L., Laoutid F., Aguedo M., Richel A., Brohez S., Delvosalle C., Dubois P. (2016). Phosphorus and nitrogen derivatization as efficient route for improvement of lignin flame retardant action in PLA. Eur. Polym. J..

[29] Costes L., Laoutid F., Brohez S., Delvosalle C., Dubois P. (2017). Phytic acid – lignin combination : A simple and e ffi cient route for enhancing thermal and fl ame retardant properties of polylactide. Eur. Polym. J..

[30] Gordobil O., Delucis R., Egüés I., Labidi J. (2015). Kraft lignin as filler in PLA to improve ductility and thermal properties. Ind. Crop. Prod..

[31] Cayla A., Rault F., Giraud S., Salaün F., Fierro V., Celzard A. (2016). PLA with intumescent system containing lignin and ammonium polyphosphate for flame retardant textile. Polymers.

[32] Maqsood M., Seide G. (2018). Investigation of the Flammability and Thermal Stability of Halogen-Free Intumescent System in Biopolymer Composites Containing Biobased Carbonization Agent and Mechanism of Their Char Formation. Polymers.

[33] Teoh E.L., Mariatti M., Chow W.S. (2016). Thermal and Flame Resistant Properties of Poly (Lactic Acid)/Poly (Methyl Methacrylate) Blends Containing Halogen-free Flame Retardant. Procedia Chem..

[34] Wei L.L., Wang D.Y., Chen H.B., Chen L., Wang X.L., Wang Y.Z. (2011). Effect of a phosphorus-containing flame retardant on the thermal properties and ease of ignition of poly(lactic acid). Polym. Degrad. Stab..

[35] Fox D.M., Lee J., Citro C.J., Novy M. (2013). Flame retarded poly(lactic acid) using POSS-modified cellulose. 1. Thermal and combustion properties of intumescing composites. Polym. Degrad. Stab..

[36] Shabanian M., Kang N.J., Wang D.Y., Wagenknecht U., Heinrich G. (2013). Synthesis of aromatic-aliphatic polyamide acting as adjuvant in polylactic acid (PLA)/ammonium polyphosphate (APP) system. Polym. Degrad. Stab..

